# Development and validation of a nomogram model for predicting postprandial hypertriglyceridemia

**DOI:** 10.3389/fnut.2025.1622385

**Published:** 2025-10-01

**Authors:** Wei Gu, Liwei Shi, Xiaolong Li, Kunjie Zheng, Guangyao Song

**Affiliations:** ^1^Department of Endocrinology, Hengshui People's Hospital, Hengshui, China; ^2^Department of Endocrinology, Hebei Provincial People's Hospital, Shijiazhuang, China

**Keywords:** postprandial, hypertriglyceridemia, predictive modelling, risk factor, risk prediction model

## Abstract

**Objective:**

This study aims to identify risk factors associated with postprandial hypertriglyceridemia (PHTG) and develop a validated predictive model for its assessment.

**Methods:**

We recruited 346 volunteers from the outpatient clinic of Hebei Provincial People’s Hospital between January and December 2019. Participants were divided into a model group (January–September 2019, *n* = 256) and an external validation group (October–December 2019, *n* = 90). The model group was further categorized into a normal lipotolerance group (NFT, *n* = 164) and a PHTG group (*n* = 92) based on fasting triglyceride levels and 4-h postprandial triglyceride measurements. Univariate analysis was performed on general information and auxiliary test results. Predictors were selected using LASSO regression, and a nomogram model of PHTG risk was constructed via logistic regression. The model’s discriminatory ability was evaluated using the area under the curve (AUC). Calibration was assessed using the GiViTI calibration curves and the Hosmer–Lemeshow (H-L) test, while clinical utility was examined through decision curve analysis (DCA). Internal validation was performed using the Bootstrap method. The model’s predictive accuracy was validated in the external group.

**Results:**

Age, fasting glucose, plasma atherogenic index (AIP), and triglyceride-glucose index (TyG) were identified as independent predictors of PHTG. The developed nomogram model demonstrated strong discriminatory power, with an AUC of 0.894 (95% CI: 0.856–0.931) in the model group and 0.903 (95% CI: 0.842–0.964) in the validation group. The H-L test, DCA, and GiViTI calibration curves confirmed excellent model calibration, demonstrating a robust agreement between predicted and observed outcomes, thus supporting the model’s clinical utility.

**Conclusion:**

The prediction model developed in this study can serve as an effective tool for predicting PHTG and help identify the high-risk population of PHTG at an early stage.

## 1 Introduction

The prevalence of hypertriglyceridemia (HTG) has risen with improvements in living standards, reaching 10.4% according to the most recent National Health and Nutrition Examination Survey (NHANES) (1). Clinically, HTG is diagnosed based on fasting triglyceride (TG) levels, however, some individuals exhibit abnormal postprandial TG elevations despite normal fasting levels, a condition known as postprandial hypertriglyceridemia (PHTG). The pathophysiological significance of PHTG is primarily attributed to the impaired clearance of triglyceride-rich lipoproteins (TRLs), including chylomicrons and VLDL remnants, during the postprandial period. This dysfunction involves several key mechanisms: (1) reduced lipoprotein lipase (LPL) activity due to genetic variations or acquired factors; (2) overexpression of apolipoprotein C-III (apoC-III), a potent inhibitor of LPL; and (3) delayed hepatic uptake of remnant particles mediated by apolipoprotein E (apoE) receptors (2–4). These metabolic abnormalities lead to prolonged circulation of atherogenic TRL remnants, which can infiltrate the arterial intima, promote foam cell formation, and trigger pro-inflammatory responses—all critical steps in atherogenesis (5, 6). Several prospective studies have demonstrated that elevated non-fasting serum TG levels increase the risk of atherosclerosis, ischemic stroke, and are an independent risk factor for coronary artery disease (7–10). Since individuals spend the majority of their time in a postprandial state, early detection and intervention for PHTG are crucial. However, the lipid tolerance test remains underdeveloped in clinical practice, with issues such as non-standardized high-fat meals and lengthy examination times. Given these pathophysiological mechanisms and the technical limitations of current diagnostic approaches, there is an urgent need to develop more efficient and standardized methods for PHTG identification. The development of a machine learning-based predictive model represents a scientifically rational approach because: (1) ML algorithms can integrate multiple clinical and biochemical variables that collectively reflect the complex pathophysiology of TRL metabolism; (2) they can identify non-linear relationships and interactions among risk factors that traditional statistical methods might miss; and (3) they offer the potential for developing personalized risk assessment tools that account for the multifactorial nature of PHTG (11, 12). Machine learning (ML) algorithms represent a novel approach to constructing predictive models for disease onset and progression (13). In this study, the PHTG population was screened out through the high-fat meal test in the healthy population to identify the independent risk factors for its occurrence. The ML method was applied to establish a predictive model, and the model was internally and externally validated, with the expectation that the application of this predictive model in clinical practice can enable earlier detection or early warning of PHTG and provide active lifestyle intervention and treatment. Avoiding the adverse outcomes thus caused plays an important role of moving the port forward in chronic disease management.

## 2 Materials and methods

### 2.1 Study design

**Figure 1:**
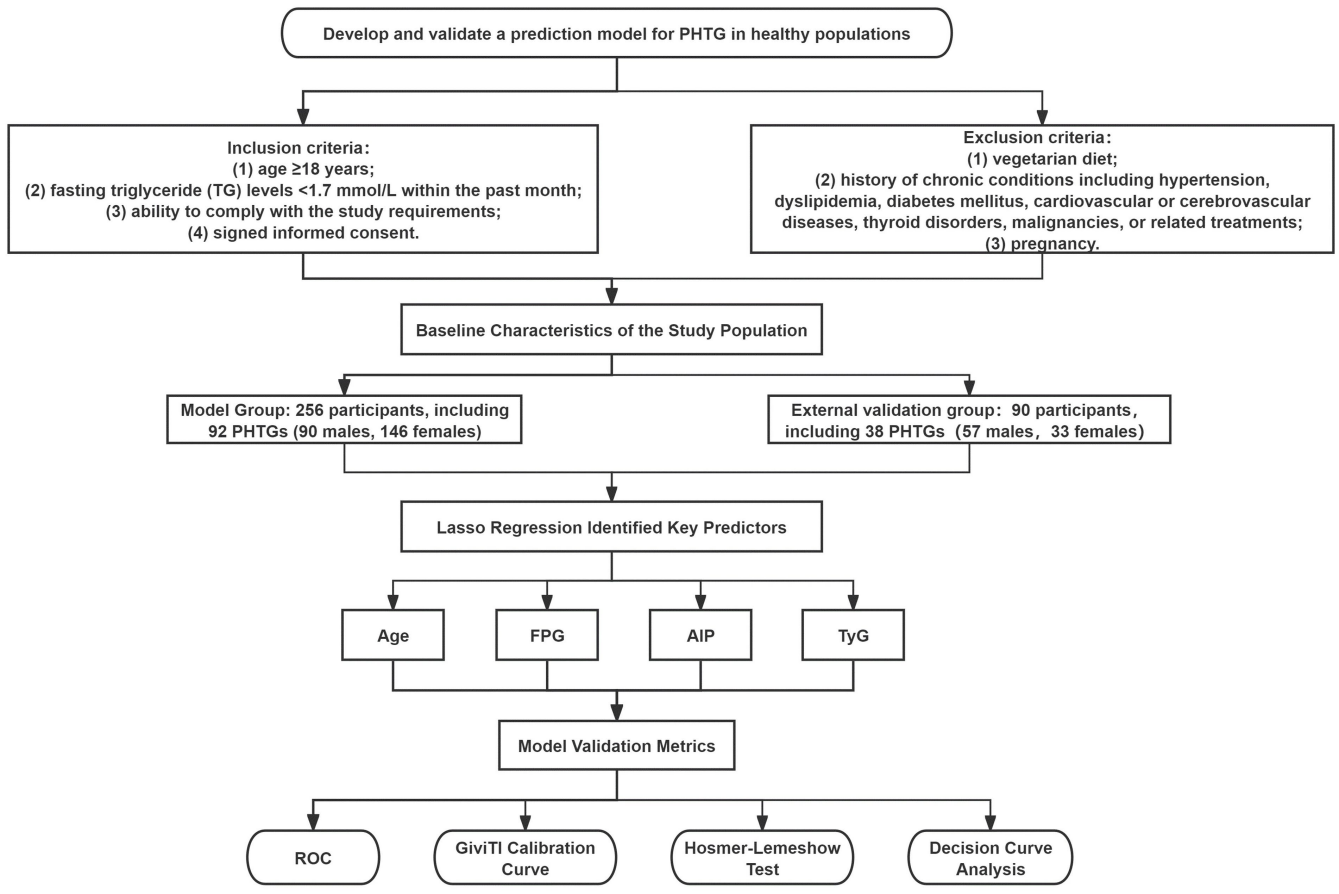
Flow chart of the study.

Volunteers were recruited from the outpatient clinic of the Department of Endocrinology at Hebei Provincial People’s Hospital between January and December 2019. Inclusion criteria: (1) age ≥18 years; (2) fasting triglyceride (TG) levels <1.7 mmol/L within the past month; (3) ability to comply with the study requirements; and (4) signed informed consent. Exclusion criteria: (1) vegetarian diet; (2) history of chronic conditions including hypertension, dyslipidemia, diabetes mellitus, cardiovascular or cerebrovascular diseases, thyroid disorders, malignancies, or related treatments; (3) pregnancy. The final analysis included 256 participants (90 males and 146 females). The sample size was determined based on a power calculation conducted prior to participant recruitment. Using GPower software (version 3.1.9.7), with an effect size of 0.3, alpha error probability of 0.05, and power of 0.90, the minimum required sample size was estimated to be 210 participants. To account for potential attrition and missing data, we increased the target sample size by 20%, resulting in a final target of 252 participants. Our enrolled sample of 256 participants therefore meets and slightly exceeds this requirement, ensuring adequate statistical power for the primary analyses. The external validation group consisted of 90 participants, including 57 males and 33 females. The model group was divided into the NFT group (*n* = 164) based on the 2019 Expert Panel Statement on PHTG (14), with fasting TG < 1.7 mmol/L and postprandial TG at 4 h < 2.5 mmol/L, and the PHTG group (*n* = 92), with fasting TG < 1.7 mmol/L but postprandial TG at 4 h ≥ 2.5 mmol/L (Figure 1). The study adhered to the Declaration of Helsinki and was approved by the Ethics Committee of Hebei Provincial People’s Hospital (approval number: 2018 no. 2). The study was registered with the China Clinical Trial Registry (registration number: ChiCTR1800019514).

### 2.2 Data collection

Two professional doctors collected and organized basic data such as weight, waist circumference (WC), body mass index (BMI), systolic blood pressure (SBP), and diastolic blood pressure (DBP), and the hospital’s physical examination center drew blood to test indicators such as blood lipids, blood sugar, and liver function.

### 2.3 High-fat meal tolerance test

Eligible volunteers received dietary guidance to avoid high-calorie and high-fat foods for 1 week prior to the high-fat meal tolerance test. The night before the test, participants were instructed to avoid oily foods for dinner and to refrain from drinking water after 22:00. On the morning of the test, volunteers fasted overnight and arrived at the hospital between 7:00 and 8:00 for fasting blood collection. Subsequently, they consumed a high-fat meal within 10 min. The meal, consisting of 1,500 kcal with a carbohydrate:fat:protein ratio of 2:6:2, was prepared based on previous study protocols (15). Volunteers were permitted to drink water during the test, and the time of meal initiation was recorded. Blood samples were collected again 4 h post-meal. During the test, participants were instructed not to consume any other food and to avoid strenuous physical activity.

### 2.4 Laboratory tests

Biochemical assays were performed using the Hitachi 7600 automatic biochemical analyzer (Hitachi, Japan). Fasting blood glucose (FBG) was measured using the glucose oxidase method, total cholesterol (TC) via the CHOD-PAP method, triglycerides (TG) using the GPO-PAP method, and low-density lipoprotein cholesterol (LDL-C) and high-density lipoprotein cholesterol (HDL-C) via the direct peroxidase method. Liver enzymes, including alanine transaminase (AST), aspartate transaminase (ALT), and gamma-glutamyl transpeptidase (GGT), were also assessed.

### 2.5 Calculation of lipid-related indices in the fasting state

The triglyceride-glucose index (TyG) was calculated as: TyG = Ln [TG (mg/dL) × FBG (mg/dL)/2]. The visceral adiposity index (VAI) was calculated as: For males: VAI = WC(cm)/[39.68 + 1.88 × BMI (kg/m^2^)] × [TG (mmol/L)/1.03] × [1.31/HDL-C (mmol/L)]; For females: VAI = WC (cm)/[36.58 + 1.89 × BMI (kg/m^2^)] × [TG (mmol/L)/0.81] × [1.52/HDL-C (mmol/L)]. The atherogenic index of plasma (AIP) was calculated as: AIP = log [TG (mmol/L)/HDL-C (mmol/L).

### 2.6 Statistical methods

Data analysis and model construction were performed using SPSS 26.0 and R Studio 2024.12.0 + 467 software. Normality of the data was assessed using the Shapiro–Wilk test (*p* > 0.05 indicating normal distribution). Normally distributed variables were expressed as mean ± standard deviation (Mean ± SD), and group comparisons were performed using the independent samples *t*-test. Non-normally distributed data were expressed as median (interquartile range) [M (IQR)], and differences between groups were analyzed using the Mann–Whitney U test. Categorical data were presented as counts (%), and comparisons between groups were conducted using the chi-square test for unordered categorical data or the rank sum test for ordered categorical data.

Least Absolute Shrinkage and Selection Operator (LASSO) regression was employed to select predictors and determine the optimal *λ* value. Logistic regression (LR) was applied using the Bootstrap method (with 1,000 iterations) to establish a predictive model. Based on the results of the logistic regression, column-line graphs for predicting PHTG were constructed (16, 17).

The discriminatory ability of the predictive model was evaluated by the area under the receiver operating characteristic (ROC) curve (AUC). Model calibration was assessed using GiViTI calibration curves and the Hosmer–Lemeshow (H-L) test, which compares the predicted probabilities to actual observations. A *p*-value < 0.05 indicated significant deviation between the predicted and observed values, suggesting poor model calibration. Conversely, a *p*-value ≥ 0.05 indicated good calibration. Decision curve analysis (DCA) was used to assess the clinical utility of the model (18).

The model was further validated using an external validation dataset, and the AUC was calculated from the ROC curve. Model consistency and clinical utility were assessed through a combination of the GiViTI calibration curve, the H-L test, and the DCA.

## 3 Result

### 3.1 Comparison of clinical data between the NFT and PHTG groups in the model group

Comparison of clinical parameters, including age, gender, BMI, WC, ALT, GGT, ALP, TC, TG4h, HDL-C, LDL-C, FBG, VAI, TyG, AIP, SBP, and DBP between the NFT and PHTG groups showed statistically significant differences (*p* < 0.05). However, the differences in AST levels between the two groups were not statistically significant (*p* > 0.05) (Table 1).

**Table 1:** Comparison of clinical data between the two groups of patients in the modeling group.

Variant	NFT group (*n* = 164)	PHTG group (*n* = 92)	*t* or *z* or *x* ^2^
Age	30.00 (26.00, 52.00)	51.00 (35.25, 58.75)	4.598**
Gender	48.00 (29.3%)	42.00 (35.2%)	8.682*
BMI	23.50 (20.70, 26.08)	25.50 (23.58, 27.50)	4.251**
WC	80.11 ± 12.10	86.87 ± 9.34	4.637**
SBP	118.80 ± 14.67	125.92 ± 15.18	3.679**
DBP	74.43 ± 9.11	77.01 ± 9.35	2.158*
FPG	5.21 ± 0.50	5.40 ± 0.52	2.754*
TG	0.84 (0.67, 1.02)	1.29 (1.08, 1.50)	9.718**
TG4h	1.54 (1.17, 1.94)	3.27 (2.81, 3.80)	13.271**
HDL-C	1.38 ± 0.31	1.24 ± 0.26	3.669**
LDL-C	2.63 (2.18, 3.13)	2.93 (2.58, 3.56)	3.873**
TC	4.33 (3.70, 4.94)	4.55 (4.13, 5.34)	2.758*
TyG	8.15 ± 0.32	8.60 ± 0.25	12.450**
VAI	1.10 ± 0.46	1.71 ± 0.51	9.803**
AIP	−0.20 ± 0.17	0.02 ± 0.13	11.766**
ALT	14.00 (10.00, 20.00)	17.00 (13.00, 23.00)	2.380*
AST	19.00 (17.00, 22.00)	19.50 (17.00, 23.00)	1.043
GGT	14.00 (11.00, 20.00)	19.00 (14.00, 26.00)	4.164**
ALP	64.42 ± 17.01	70.50 ± 18.98	2.631*

*p < 0.05, **p < 0.01; data in the table are expressed as cases (%), M (P25, P75), or $\bar{x}\pm s$ .

### 3.2 Screening of predictors for PHTG in a healthy population

**Figure 2:**
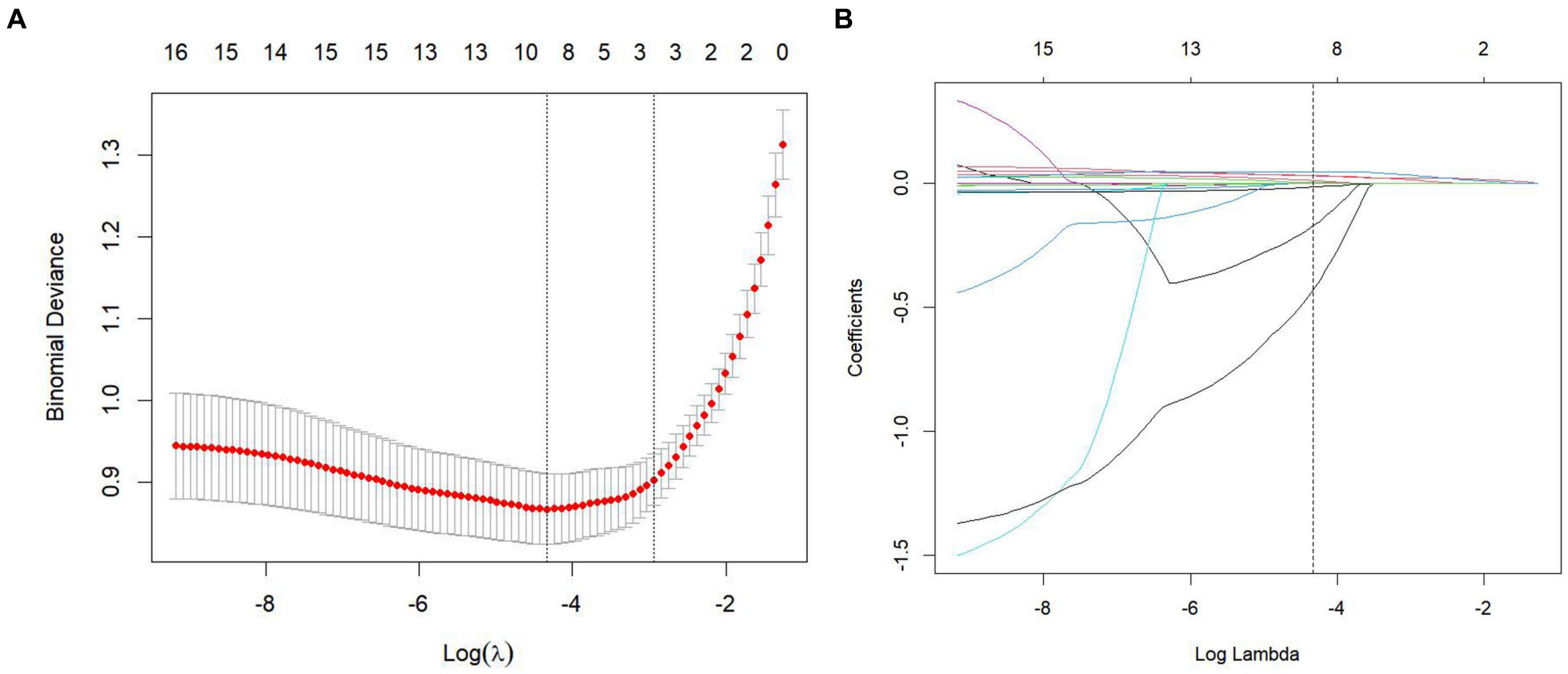
Risk factor screening for PHTG in a healthy population. **(A)** Lasso regression model cross-validation plot. **(B)** Plan of predictor coefficients.

To minimize the impact of multicollinearity among variables, 17 variables with statistically significant differences in the univariate analysis were included in the LASSO regression (Figure 2A). Ten-fold cross-validation was applied, resulting in the identification of eight clinically significant predictors: gender, age, SBP, ALT, GGT, FPG, TyG, and AIP (Figure 2B).

### 3.3 Logistic regression model for predicting PHTG in a healthy population

**Figure 3:**
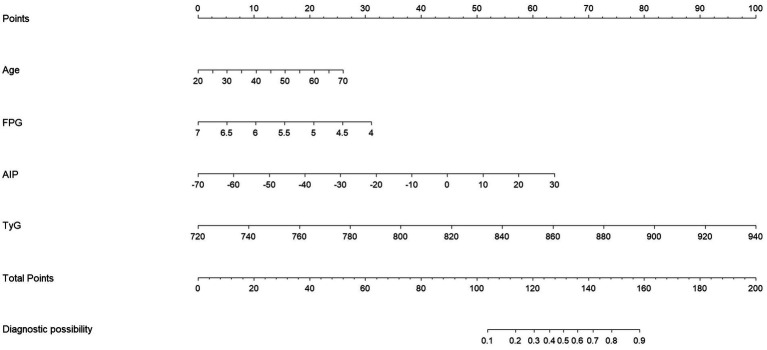
PHTG early prediction model nomogram.

Eight clinical variables—gender, age, SBP, ALT, GGT, FPG, TyG, and AIP—were included in the logistic regression model. Based on the results of multivariate analysis (Table 2), variables with *p* < 0.05 were selected for inclusion in the model, resulting in the following logistic regression equation: Logit(P) = −31.769 + 0.041 × Age − 0.910 × FPG + 0.048 × AIP + 0.040 × TyG. The predictive model for PHTG occurrence in a healthy population was represented as a nomogram. Scores for each predictor were assigned according to specific scales on the column-line diagram, which are associated with their respective risk factors. These individual scores were then summed to generate an overall score, used to estimate the likelihood of PHTG. The overall score ranges from 0 to 200, with associated risk levels varying between 10% and 90%. An elevated overall score indicates a higher risk of PHTG (Figure 3).

**Table 2:** Results of logistic regression analysis for predicting PHTG in a healthy population.

Variant	*β*	SE	*p-*value	OR	95% CI
Gender	−0.416	0.439	0.343	0.660	0.317–1.353
Age	0.041	0.014	0.004	1.042	1.018–1.067
SBP	0.009	0.013	0.486	1.009	0.988–1.030
ALT	−0.038	0.020	0.055	0.963	0.930–0.991
GGT	0.031	0.023	0.185	1.032	0.994–1.074
FPG	−0.910	0.424	0.032	0.403	0.197–0.799
AIP	0.048	0.022	0.031	1.049	1.012–1.089
TyG	0.040	0.012	0.001	1.041	1.021–1.063

### 3.4 Assessing the discriminative ability and consistency of the model

**Figure 4:**
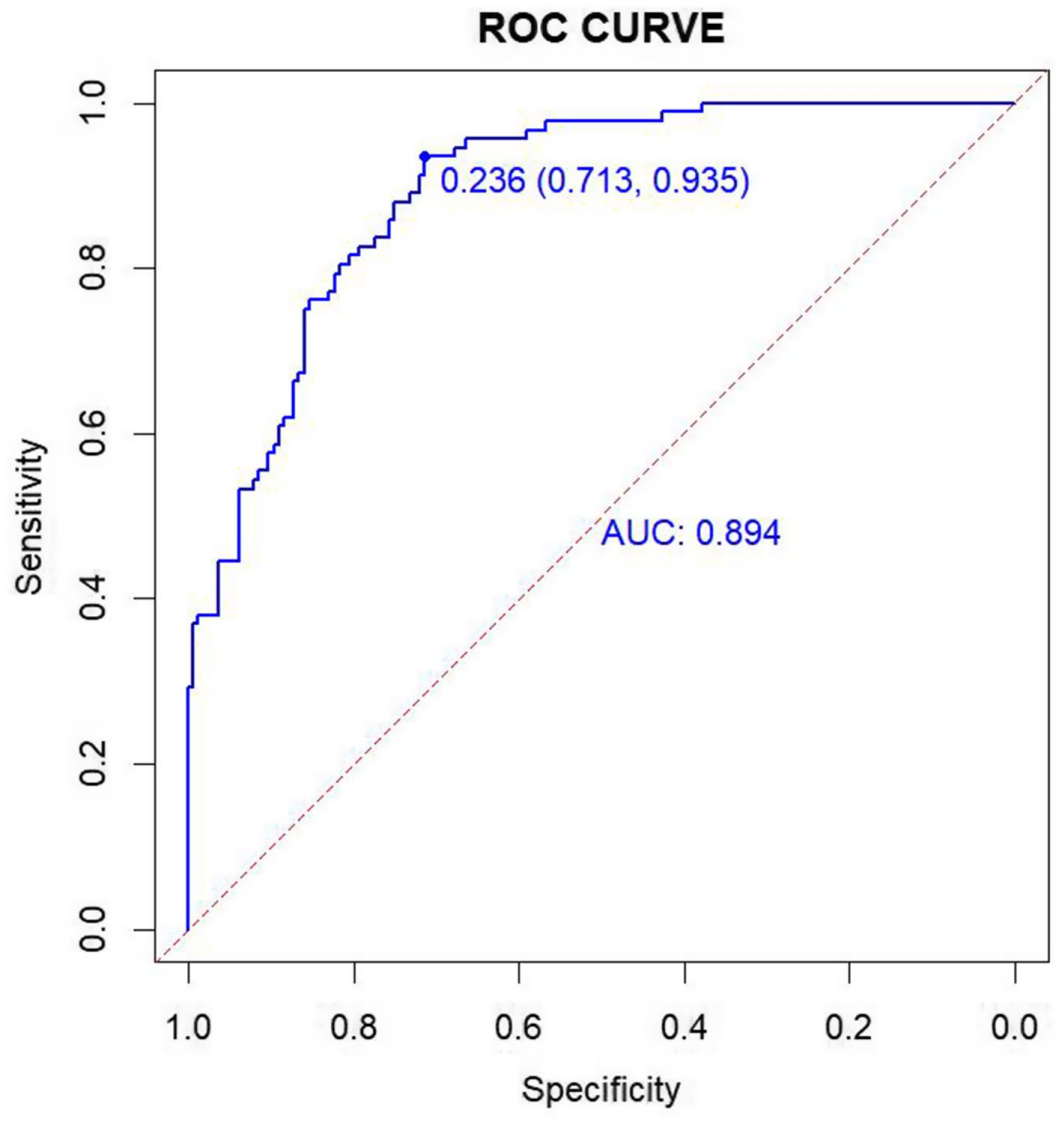
ROC curves for the modeling group.

The discriminative ability of the logistic regression model was evaluated using the ROC curve, resulting in an AUC of 0.894 (95% CI: 0.856–0.931). The model demonstrated a Youden’s index of 64.8%, a sensitivity of 71.3%, a specificity of 93.5%, and an optimal cutoff value of 0.236 (Figure 4). Calibration of the model was assessed using the Hosmer–Lemeshow goodness-of-fit test, which yielded a Chi-square value of 11.308, with 8 degrees of freedom (df) and a *p*-value of 0.1849 (*p* > 0.05). These results suggest that the model exhibits good predictive ability and high diagnostic value for predicting the occurrence of PHTG in a healthy population.

### 3.5 Assessing the clinical utility of the model

**Figure 5:**
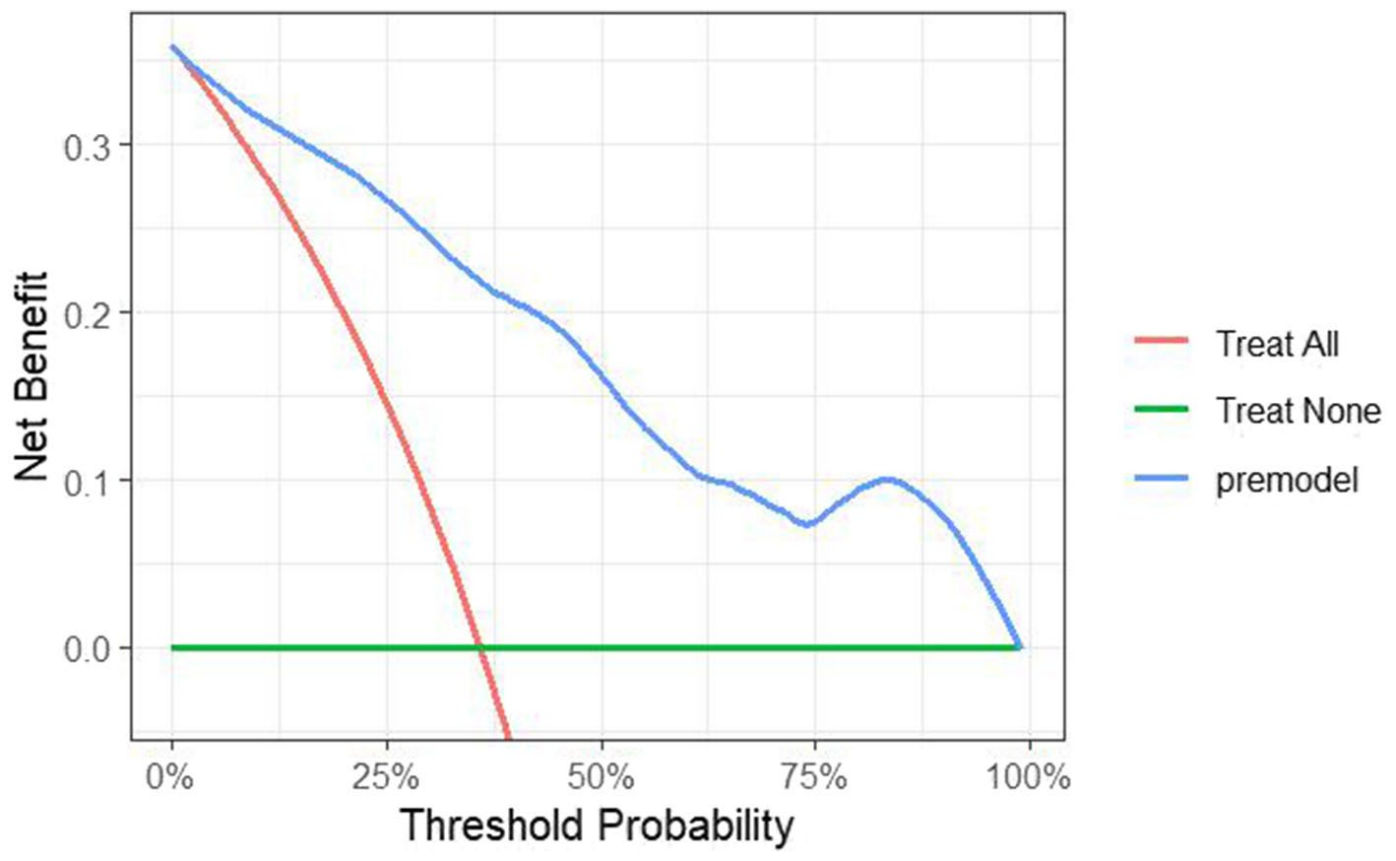
DCA curves for establishing a predictive model for the occurrence of PHTG in the healthy population of the model group.

The DCA for the model was plotted. When the threshold probability exceeded 0, the model curve was positioned above the two extreme value lines (Figure 5), indicating that the model predicts the benefits of timely clinical interventions. This suggests that the model holds good clinical value in guiding interventions for patients at risk of PHTG.

### 3.6 Internal validation of the model

**Figure 6:**
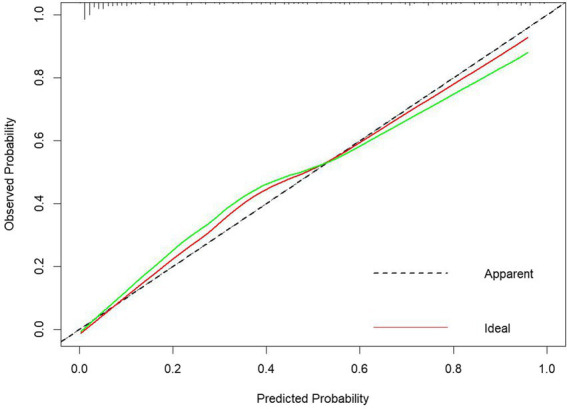
GiViTI calibration curves for modeling group logistic regression models.

The model was internally validated using the Bootstrap method with 1,000 repeated samplings. The mean area AUC obtained from these samplings was 0.900 (95% CI: 0.841–0.920), with an AUC greater than 0.7, indicating that the model effectively differentiates the PHTG population within the healthy population. The GiViTI calibration curve, shown in Figure 6, demonstrates that the model maintains high accuracy after calibration. The calibration curves indicate that the original and calibrated curves closely align, both effectively predicting PHTG in the healthy population (Figure 6).

### 3.7 External validation of the model

**Figure 7:**
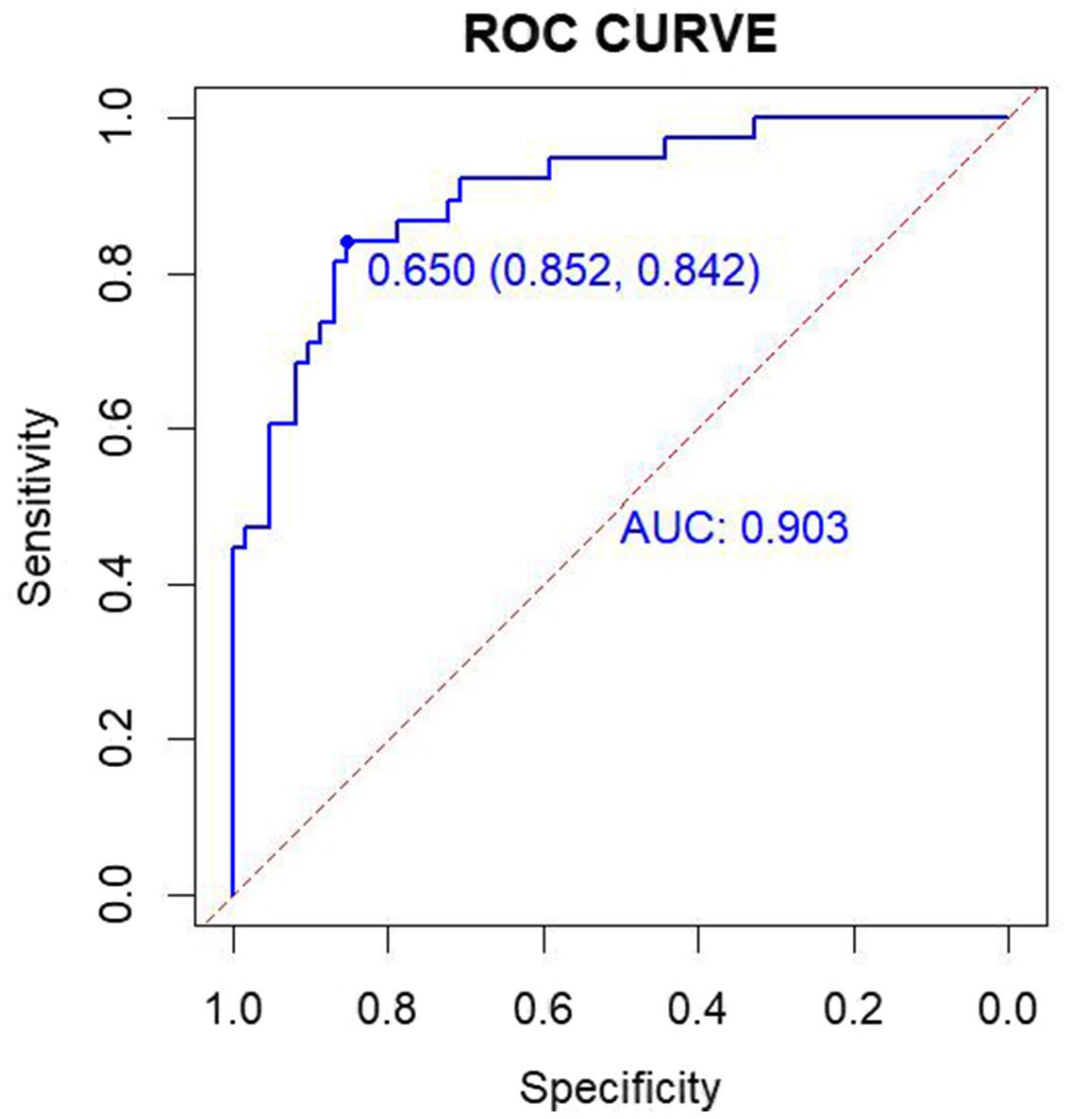
ROC curve for external validation group.

**Figure 8:**
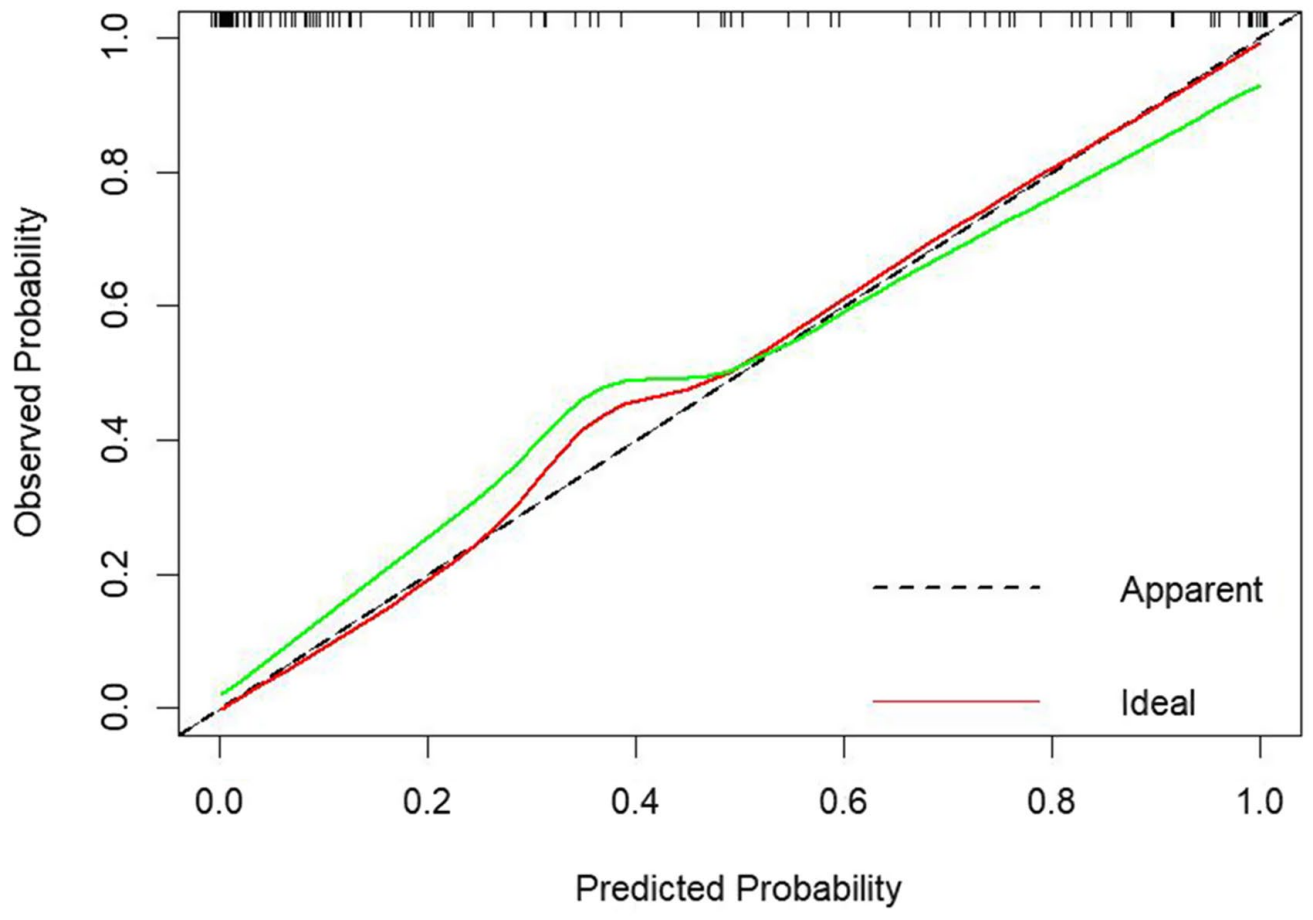
GiViTI calibration curves of logistic regression model for external validation group.

**Figure 9:**
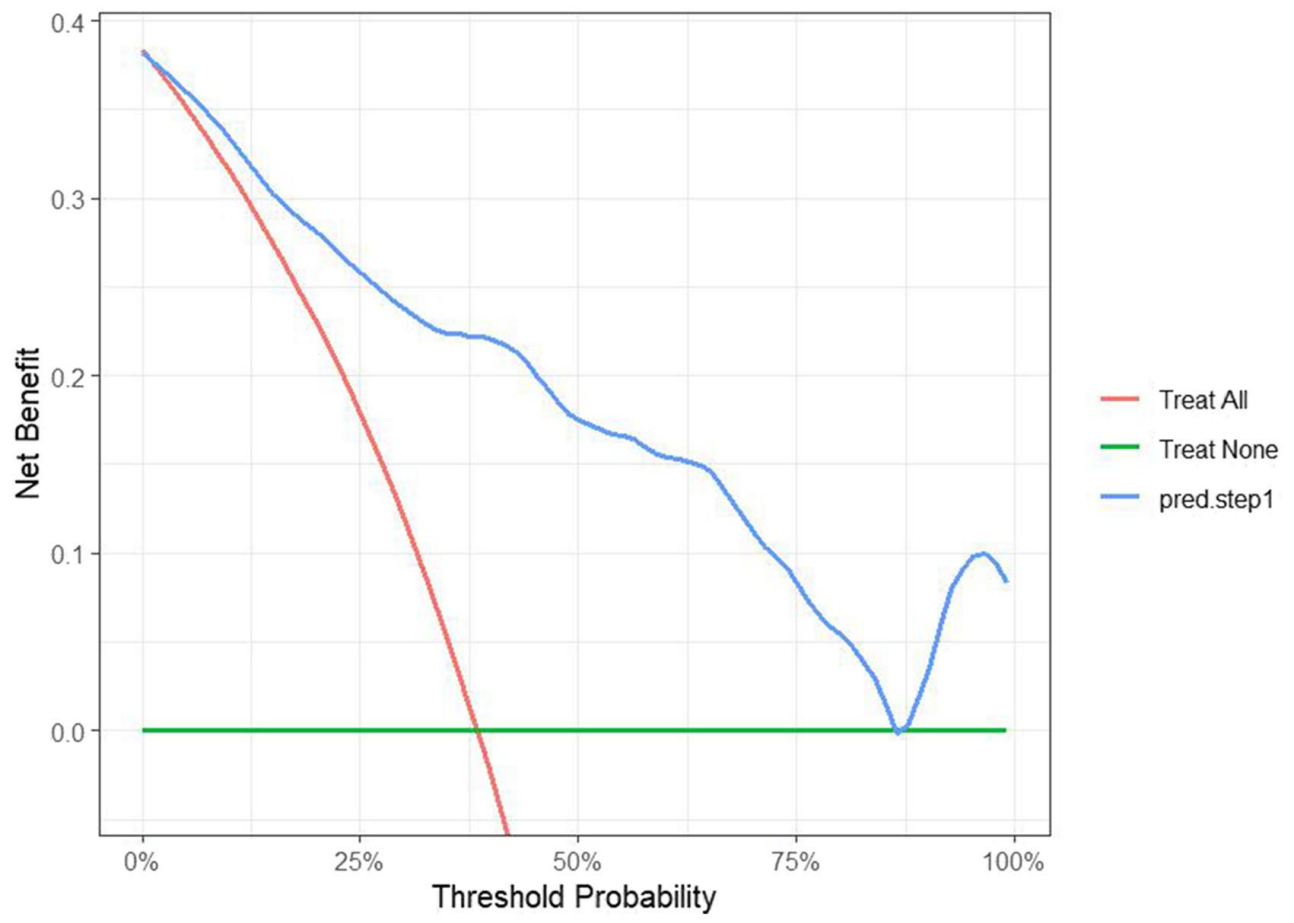
DCA curves of the predictive model for the occurrence of PHTG in the healthy population of the external validation group.

The data from the external validation group were applied to the previously constructed logistic regression model to calculate the risk values for the occurrence of PHTG in the healthy population. Based on a critical risk threshold of 0.389, individuals in the validation group were classified as at risk for PHTG if their risk value was ≥0.236, and not at risk if the risk value was <0.236. The model’s discriminatory ability and calibration were evaluated using the ROC curve and the Hosmer–Lemeshow goodness-of-fit test. The results showed an AUC of 0.903 (95% CI: 0.842–0.964), with a sensitivity of 85.2%, specificity of 84.2%, and a Youden’s index of 69.4% (Figure 7). The Hosmer–Lemeshow test showed a Chi-square value of 13.326, with 8 degrees of freedom (df) and a *p*-value of 0.101 (*p* > 0.05). These results indicate that the model has strong predictive power and high diagnostic value for PHTG occurrence in the healthy population. The GiViTI calibration curve, shown in Figure 8, demonstrates that the original curve aligns closely with the calibrated curve, suggesting both models effectively predict PHTG in the healthy population. Additionally, the DCA curve, shown in Figure 9, suggests that the model provides clinically meaningful predictions of PHTG risk, with timely clinical interventions being beneficial.

## 4 Discussion

PHTG refers to a metabolic state characterized by abnormally elevated TG levels following a meal, reflecting the body’s insufficient ability to regulate lipid metabolism after fat intake. PHTG is closely associated with insulin resistance and visceral fat accumulation (19). Following a meal, TG levels gradually increase, peak at 3–4 h, and slowly return to baseline within 6–8 h (20). Given that most of the day is spent in the postprandial state, with a relatively short fasting period, individuals are frequently exposed to the postprandial TG cycle. Non-fasting/postprandial TG levels are independent risk factors for coronary artery disease, stroke (7), and all-cause mortality in type 2 diabetes (8). Elevated postprandial TG accelerates atherosclerosis progression by promoting endothelial dysfunction, LDL oxidation, and inflammatory responses (21). PHTG induces chronic inflammation in adipocytes through lysosomal dysfunction triggered by TG-rich lipoproteins and impaired autophagic flow in an mTOR-dependent manner (9). Moreover, PHTG is associated with increased hepatic VLDL overproduction and lipoprotein lipase dysfunction (22). Consequently, early detection and intervention for PHTG are critical. The assessment and diagnosis of PHTG typically rely on the lipid tolerance test, but this approach is not widely used in clinical practice for several reasons: (1) the test requires blood sample collection after consuming a high-fat diet, followed by dynamic postprandial lipid assessments, which is complex, time-consuming, and difficult for patients to adhere to; and (2) there is no standardized high-fat meal, and the fat content and calorie values in study meals vary, complicating quality control. Therefore, how to assess the risk of PHTG through more convenient methods has become a research hotspot. From a clinical standpoint, the challenges in implementing lipid tolerance tests limit the early identification of individuals with PHTG, who remain undiagnosed under current fasting-based screening protocols. This gap is particularly concerning given that postprandial dyslipidemia contributes to residual cardiovascular risk even in statin-treated patients (23). Clinicians are often faced with patients who have normal fasting lipids yet present with premature atherosclerosis, suggesting underlying postprandial abnormalities. Therefore, a practical and reliable tool for identifying PHTG could significantly enhance risk stratification and allow timely intervention through lifestyle modification or pharmacological treatments such as fibrates or omega-3 fatty acids, which specifically target postprandial hypertriglyceridemia (24).

Recent advancements in lipid metabolism research have led to the use of lipid-related indices as practical and effective tools for predicting and screening metabolic syndrome, insulin resistance, and cardiovascular diseases (25, 26). Our previous study identified significant increases in the TyG, VAI, and AIP in PHTG patients, all of which were positively correlated with fasting TG and 4-h TG levels after a high-fat meal. These indices showed high predictive value for PHTG. The TyG index, proposed by Guerrero-Romero in 2010 (27), assesses insulin resistance by combining fasting TG and glucose levels. It is highly correlated with the “gold-standard” insulin clamp test and can predict the risk of metabolic syndrome and type 2 diabetes (28). In the context of PHTG, the TyG index serves as an important reference for early screening, reflecting the impact of insulin resistance on lipid metabolism. In this study, incorporating TyG provided the model with multidimensional information on visceral lipid metabolism, significantly enhancing its ability to assess PHTG risk. The VAI, developed by Amato in 2010 (29), is a composite index combining waist circumference, BMI, TG, and HDL-C, initially used as a surrogate for visceral obesity (30). It has since been shown to be associated with insulin resistance, metabolic syndrome, cardiovascular risk (31), and the development of non-alcoholic fatty liver disease (NAFLD), with high VAI levels increasing NAFLD risk (32). The AIP, proposed by Dobiásová in 2000 (33), reflects lipid metabolism disorders by assessing both elevated TG and decreased HDL-C levels, serving as a marker for atherosclerosis (34). Studies have shown that AIP is positively correlated with the proportion of small, dense LDL particles, a major risk factor for atherosclerosis (35). AIP is widely used to assess cardiovascular risk in patients with coronary heart disease, chronic kidney disease, and metabolic syndrome (36–38). The integration of these indices into a nomogram model offers a clinician-friendly tool that can be readily applied in outpatient settings without requiring postprandial testing. For example, a patient with elevated TyG and AIP values—even in the presence of normal fasting TG—should raise suspicion of PHTG and prompt further evaluation or preventive measures. This approach aligns with recent guidelines emphasizing non-traditional risk markers for comprehensive cardiovascular risk assessment (39). Moreover, identifying high-risk individuals early allows for tailored interventions, such as dietary counseling focused on low-glycemic and low-fat intake, which has been shown to ameliorate postprandial lipemia (40).

In this study, logistic regression analysis of variables such as age, gender, SBP, ALT, GGT, FPG, TyG, and AIP revealed significant differences between the PHTG and normal lipid groups. A risk nomogram model was developed, including age, FPG, TyG, and AIP as predictors. The model was internally and externally validated, demonstrating excellent differentiation ability, with an AUC of 0.894 (95% CI: 0.856–0.931) for the model group and 0.903 (95% CI: 0.842–0.964) for the validation group. The GiViTI calibration curve and Hosmer–Lemeshow test confirmed that the model had good calibration and consistency, suggesting its potential for predicting PHTG risk in healthy populations. The DCA indicated high clinical utility, making it valuable for screening high-risk patients and aiding in the prevention and treatment of PHTG.

Limitations: (1) The study lacks multicenter data, and the overall sample size is small, highlighting the need for larger studies; (2) The outcome variable for PHTG was not dichotomized (i.e., progressed to PHTG vs. not progressed), and future research should stratify this variable; (3) The study design is retrospective, which may introduce bias. Future prospective validation studies with extended follow-up are planned to improve the model.

## 5 Conclusion

This study developed a predictive model for PHTG designed to help distinguish affected individuals from those with normal lipid levels. As a practical alternative to more complex lipid tolerance tests, this tool may support early identification and personalized intervention in high-risk populations, potentially contributing to improved preventive strategies for PHTG-related cardiometabolic diseases.

## Data Availability

The original contributions presented in the study are included in the article/supplementary material, further inquiries can be directed to the corresponding author.
